# Supplementary material for: Impact of occluder device configurations in in-silico left atrial hemodynamics for the analysis of device-related thrombus
Source: PLoS Comput Biol. 2024 Sep 26;20(9):e1011546. doi: 10.1371/journal.pcbi.1011546 (PMC11460709; doi:10.1371/journal.pcbi.1011546)
Supplement: S1 Text — Section A: Spring-based model definition. Section B: Discrete phase model effects on blood flow behaviour. (PDF) [file pcbi.1011546.s001.pdf]

A Supporting Information

Section A:Spring-based model definition

Time-resolved cardiac computed tomography scans were not included for the analysis of the study cohort. Previous literature [31, 36, 49] has underscored the significance of left atrial (LA) wall motion, not only in understanding blood flow dynamics but also for adhering to the principles of mass conservation in the in-silico simulations.

Therefore, a spring-based dynamic mesh approach from ANSYS Fluent 2022 (ANSYS Inc, USA) was applied to the LA meshes. Clinically, this motion represents the passive displacement of the mitral valve annulus ring based on the ventricular contraction during systolic phase. This occurs without radial LA motion, assuming that the atrial fibrillation (AF) patients do not have strong active contraction.

The displacements of the LA wall are described by the following equation [50]:

$$\overrightarrow{x}^{n+1} = \overrightarrow{x}^{n} + \beta \Delta \overrightarrow{x}^{m,converged}_{spring}, \tag{12}$$

where $\overrightarrow{x}^{n+1}$ and $\overrightarrow{x}^{n}$ represent the positions at the next time step and the current time step, respectively. β denotes the node relaxation set to 1 which indicates no under-relaxation. $\overrightarrow{x}^{m,converged}_{spring}$ refers to the iterative equation iterated until the node displacements reach the equilibrium state, where the net force on a node due to all connected springs is zero:

$$\Delta \overrightarrow{x}^{m+1}_{i} = \frac{\sum_j^{n_i} k_{ij} \Delta \overrightarrow{x}^{m}_{j}}{\sum_j^{n_i} k_{ij}} \tag{13}$$

Moreover, to control and tune the solution of the equation, we established a convergence tolerance of 0.0001. Additionally, we set a limit of 200 iterations to achieve equilibrium and employed a spring constant factor k of 0.45. This factor signifies a balance between creating new cells ($k = 1$) and restoring the original mesh distribution ($k = 0$). Due to the boundary constraints of the left atrial elements adjacent to the surface plane defining the mitral valve motion, we allowed for some degrees of freedom in the model. However, to prevent the generation of degenerate elements, a remeshing process is also implemented within the Ansys Fluent solver. This process involves local cell, local face, and region face adjustments. Consequently, changes in mesh resolution of 3-6% were observed.

The imposed longitudinal dynamic mesh displacements at the mitral valve plane were previously synchronized based on the cardiac rhythm extracted from the ECG and the echocardiographic Doppler measurement of the mitral valve.

Section B:Discrete phase model effects on blood flow behaviour

A preliminary analysis was conducted to observe any possible effects of the DPM introduction in the CFD solver on blood flow behavior. Hence, two scenarios were defined per case in two patients, introducing the DPM to interact with the LA domain fluid solution and without introducing it.

Analysing simulation outcomes in both cases, it was found that the inclusion of the DPM did not cause a noteworthy impact on the flow behavior that could alter the interpretation or estimation of DRT. Specifically, the averaged velocities at the device surface did not significantly changed throughout the cardiac beat (0.0574 $\pm$ 0.0355 m/s with DPM inclusion and 0.0599 $\pm$ 0.0264 m/s without DPM inclusion). However, a minor sustained velocity in the systolic phase and an increase in the diastolic peak were observed with the introduction of the DPM model (see S1 Fig graph). Qualitatively, similar flow patterns were observed in both patients (see S1 Fig). At the beginning of diastole (t = 0.3 s), laminar flow with velocities higher than the 0.2 m/s threshold were

predominant, except in the central part of the disk with velocities of 0.12 m/s for the no DPM inclusion scenario and 0.14 m/s in the DPM scenario. Blood flow at low velocities and with recirculations in the center of the device were observed during the end of diastole (t = 0.7 s) in both cases, slightly lower without the DPM inclusion.
