# Supplementary figures and images for: Impact of occluder device configurations in in-silico left atrial hemodynamics for the analysis of device-related thrombus
Source: PLoS Comput Biol. 2024 Sep 26;20(9):e1011546. doi: 10.1371/journal.pcbi.1011546 (PMC11460709; doi:10.1371/journal.pcbi.1011546)

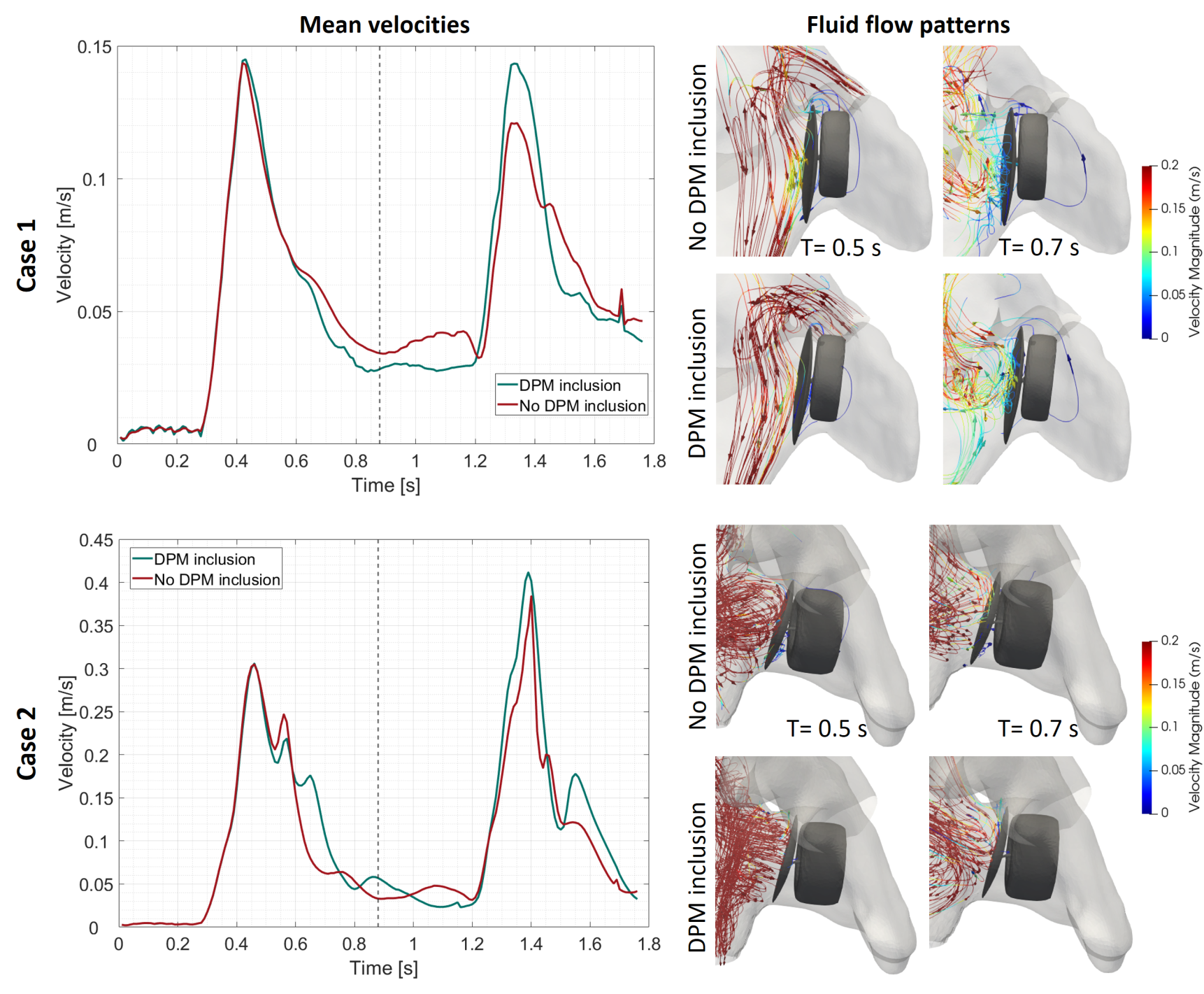

Supplement: S1 Fig — The left side shows a quantitative comparison of the average velocities close to the device surface within 2 cardiac cycle beats, with red indicating simulations without DPM inclusion and blue indicating simulations with DPM inclusion. The right side shows simulated blood flow patterns during early- (t = 0.5 s) and late-diastole (t = 0.7 s). (TIFF) [file pcbi.1011546.s002.tiff]
